# Characterizing Errors in Pharmacokinetic Parameters from Analyzing Quantitative Abbreviated DCE-MRI Data in Breast Cancer

**DOI:** 10.3390/tomography7030023

**Published:** 2021-06-23

**Authors:** Kalina P. Slavkova, Julie C. DiCarlo, Anum S. Kazerouni, John Virostko, Anna G. Sorace, Debra Patt, Boone Goodgame, Thomas E. Yankeelov

**Affiliations:** 1 Department of Physics, The University of Texas at Austin, Austin, TX 78712, USA; kslav@utexas.edu; 2 Livestrong Cancer Institutes, The University of Texas at Austin, Austin, TX 78712, USA; julie.dicarlo@utexas.edu (J.C.D.); jack.virostko@austin.utexas.edu (J.V.); 3 Oden Institute for Computational Engineering and Sciences, The University of Texas at Austin, Austin, TX 78712, USA; 4 Department of Radiology, The University of Washington, Seattle, WA 98195, USA; anumkaz@uw.edu; 5 Department of Diagnostic Medicine, The University of Texas at Austin, Austin, TX 78712, USA; 6 Department of Radiology, The University of Alabama at Birmingham, Birmingham, AL 35294, USA; asorace@uabmc.edu; 7 Department of Biomedical Engineering, The University of Alabama at Birmingham, Birmingham, AL 35294, USA; 8 O’Neal Comprehensive Cancer Center, The University of Alabama at Birmingham, Birmingham, AL 35294, USA; 9 Texas Oncology, Austin, TX 78731, USA; Debra.Patt@usoncology.com; 10 Seton Hospital, Austin, TX 78712, USA; bwGoodgame@ascension.org; 11 Department of Internal Medicine, The University of Texas at Austin, Austin, TX 78712, USA; 12 Department of Imaging Physics, The University of Texas MD Anderson Cancer Center Houston, Houston, TX 77030, USA; 13 Department of Oncology, The University of Texas at Austin, Austin, TX 78712, USA; 14 Department of Biomedical Engineering, The University of Texas at Austin, 107 W Dean Keeton Street, Stop C0800, Austin, TX 787812, USA

**Keywords:** Patlak, Kety–Tofts, dynamic contrast-enhanced MRI, abbreviated breast MRI, quantitative MRI

## Abstract

This study characterizes the error that results when performing quantitative analysis of abbreviated dynamic contrast-enhanced magnetic resonance imaging (DCE-MRI) data of the breast with the Standard Kety–Tofts (SKT) model and its Patlak variant. More specifically, we used simulations and patient data to determine the accuracy with which abbreviated time course data could reproduce the pharmacokinetic parameters, *K^trans^* (volume transfer constant) and *v_e_* (extravascular/extracellular volume fraction), when compared to the full time course data. SKT analysis of simulated abbreviated time courses (ATCs) based on the imaging parameters from two available datasets (collected with a 3T MRI scanner) at a temporal resolution of 15 s (N = 15) and 7.23 s (N = 15) found a concordance correlation coefficient (CCC) greater than 0.80 for ATCs of length 3.0 and 2.5 min, respectively, for the *K^trans^* parameter. Analysis of the experimental data found that at least 90% of patients met this CCC cut-off of 0.80 for the ATCs of the aforementioned lengths. Patlak analysis of experimental data found that 80% of patients from the 15 s resolution dataset and 90% of patients from the 7.27 s resolution dataset met the 0.80 CCC cut-off for ATC lengths of 1.25 and 1.09 min, respectively. This study provides evidence for both the feasibility and potential utility of performing a quantitative analysis of abbreviated breast DCE-MRI in conjunction with acquisition of current standard-of-care high resolution scans without significant loss of information in the community setting.

## 1. Introduction

While X-ray mammography is the accepted screening method for the early detection of breast cancer in the general population of women, magnetic resonance imaging (MRI) of the breast has seen a recent increase in use. MRI offers superior soft tissue contrast and, as a result, greater sensitivity to suspicious lesions [1,2,3], especially with the use of a contrast agent [4,5]. Breast MRI is particularly beneficial to women who are at higher risk for breast cancer due to genetic factors and to women with dense breast tissue, as well as for pretreatment planning [6,7]. In one study of 501 women, MRI yielded a sensitivity of 91%, compared to 18% for clinical breast exam, 50% for mammography, and 52% for ultrasonography [1]. In terms of clinical outcome, MRI alone has increased the detection of lesions in the pre-invasive phase, and MRI quantitative methods as a whole have been shown to increase the diagnostic power of clinical evaluations [8,9].

One fundamental issue with breast MRI, however, is its high cost due (partly) to an examination time of approximately 40 min [10]. For this reason, there is a push to develop a so-called “abbreviated” breast examination, which aims to reduce the total amount of time that is required for imaging so that screening costs can be reduced without sacrificing diagnostic information [10,11,12]. One strategy is to limit the timing of the contrast-enhanced component of the scan to capture only the enhancement phase, which has previously been shown to differentiate between benign and malignant breast lesions [13,14]. Using this strategy, Kuhl et al. were able to achieve a sensitivity of 100% and a specificity of 94% using an abbreviated protocol that took just three minutes to acquire [10]. Another study by Grimm et al. showed that adding additional post-enhancement images did not increase the specificity of the two abbreviated protocols in question [15]. This same study found that there was no significant difference (*p* > 0.05) between the sensitivities of these two abbreviated protocols and a full protocol breast MRI exam [15].

While MRI exhibits superior sensitivity overall compared to other standard screening techniques (e.g., mammography and ultrasound), its specificity has been shown to be lower in the context of ductal carcinoma in situ (DCIS). One study by Kuhl et al. revealed that low-grade DCIS was the only cancer missed by MRI but detected by mammography [8,10,16]. False positives may lead to unnecessary biopsies with concomitant stress to the patient and cost to the healthcare system [8,16]. Additionally, due to intra-tumoral heterogeneity, the results from histological analysis of biopsy samples may be inaccurate [17]. Thus, there is substantial effort to increase the diagnostic specificity of breast MRI in the abbreviated setting and interest in the development and translation of quantitative imaging schemes [18]. Dynamic contrast enhanced MRI (DCE-MRI) is one imaging modality that can quantitatively report on properties related to tissue vascularity and volume fractions and thereby potentially add specificity to exams.

DCE-MRI is the sequential acquisition of *T_1_*-weighted images before, during, and after the injection of a gadolinium-based contrast agent. To perform quantitative DCE-MRI analysis, in addition to the time course data just mentioned, a pre-contrast *T_1_* map, an arterial input function (an estimate of the time rate of change of the concentration of the contrast agent in the blood plasma), and a pharmacokinetic model to analyze the resulting data are also required. Typical pharmacokinetic analyses include the Kety–Tofts [19], tissue homogeneity [20], reference region [21], shutter-speed [22], and Patlak models [23]. Quantitative information derived from full-length DCE-MRI acquisitions (approximately 10 min in length) has demonstrated added benefit in distinguishing malignancies [5,13,24]. Specifically, the volume transfer constant, *K^trans^*, has been shown to statistically distinguish malignant from benign lesions, including in the ultra-fast DCE-MRI setting with superior temporal resolution [13,24,25,26]. Still, quantitative DCE-MRI can be challenging to incorporate into the clinical workflow as it requires higher temporal resolution data which comes at the expense of missing spatial resolution in the images required by radiologists. This is a pronounced problem in the ultra-fast regime that lacks high spatial resolution [24] and thus does not provide a viable solution for quantitative imaging in the clinical workflow. Importantly, no studies have been published that seek to characterize the errors introduced into pharmacokinetic modeling that is performed on the shortened time course data acquired from an abbreviated protocol.

The overall goal of this contribution is to systematically determine the error that results when applying reference region and Patlak analyses on retrospectively abbreviated quantitative DCE-MRI data. More specifically, we characterize the error induced in the volume transfer rate (*K^trans^*) and the extravascular/extracellular volume fraction (*v_e_*) when we apply these models to both simulated and experimentally measured DCE-MRI data obtained in two different clinical breast imaging settings: a multi-site, network-based clinical trial and a single site, community-based imaging center. Analysis in multiple settings provides a robust approach for future feasibility and widespread implementation. For each abbreviated time course, we quantify the error in the parameter values as compared to those measured using the original, full-time course data to determine a recommendation on how quantitative analysis of abbreviated DCE-MRI of the breast can be performed in the clinical setting.

## 2. Methodology

Two DCE-MRI datasets were analyzed in this study. One was from the International Breast MR Consortium (IBMC) 6883 multi-site trial provided by the American College of Radiology Imaging Network (ACRIN) [6,13] while the other consisted of imaging data collected at a regional imaging center in the community setting in a major metropolitan city in the United States. No identifying information was associated with any patient dataset.

### 2.1. ACRIN 6883 Trial DCE-MRI Acquisition

Patients (N = 821) (often more than one lesion per patient) who enrolled in the IBMC 6883 multi-site trial were initially referred for breast biopsy due to the detection of suspicious lesions [6]. All women received a BI-RADS equivalent label for each lesion [6] and were scanned using a 1.5 T scanner equipped with a dedicated breast coil. A subset of patient datasets (N = 35) available to the authors had multi-*TR* data, collected at *TR*s of (110, 200, 300, 1200 ms), which was analyzed with the spoiled gradient echo model to compute pre-contrast *T_1_* maps for the imaged tissue. DCE-MRI data was collected with *TR*/*TE* = 100/4–5 ms, a flip angle of *α* = 90°, and an acquisition matrix of 256 × 128 over a (160–180) × (160 to 180) mm^2^ field-of-view (FOV) with a slice thickness of 4 mm. Each of the 11-slice sets was collected in 15 s over various scan times ranging from 14 total time points (3.50 min) to 37 total time points (9.25 min). The dynamic scan was initiated simultaneously with the delivery of 0.1 mmol/kg of a gadolinium chelate (Omniscan, GE Healthcare; Prohance, Bracco; or Magnevist, Berlex) administered over 10 s through a catheter placed within an antecubital vein followed by a saline flush. No arterial input function was available for this study; however, dynamic data were collected from reference regions drawn within the chest wall muscle of each patient, thereby enabling a reference region analysis. To determine the tumor regions-of-interest (ROIs), a conservative boundary was drawn around each lesion and refined by selecting voxels with a percent enhancement greater than 50% [6]. Going forward, we will refer to these data as the ACRIN dataset. The acquisition details were sourced from previous studies [6,13].

### 2.2. Single-Site DCE-MRI Acquisition

Patients (N = 22) (often a single lesion per patient) with locally advanced breast cancer were scanned prior to beginning neoadjuvant therapy using a 3 T Skyra (Siemens, Erlangan, Germany) equipped with a 16-channel receive double-breast coil (Invivo, Gainsville, FL, USA). Variable-flip angle (VFA) data was collected at flip angles of 2° to 20° in increments of 2°. This VFA data was then fit to the spoiled gradient echo (SPGR) model, implemented in MATLAB (Mathworks, Natick, MA, USA) using *B_1_*-corrected flip angles to estimate pre-contrast *T_1_* maps for the imaged tissue. The data for the *B_1_*-correction were obtained via the Siemens TurboFLASH sequence with a pre-conditioning radiofrequency pulse [27] with *TR*/*TE* = 8680/2 ms, a flip angle of *α* = 8°, an acquisition matrix of 96 × 96, and a slice thickness of 5 mm. DCE-MRI data was collected with *TR*/*TE* = 7.02/4.60 ms, a flip angle of *α* = 6°, an acquisition matrix of 192 × 192 over a 256 × 256 mm^2^ FOV, a slice thickness of 5 mm, and a GRAPPA (generalized autocalibrating partial parallel acquisition) acceleration factor of 2. Each of the 10-slice sets was collected in 7.27 s across 66 total time points for 8 min of total DCE-MRI scan time. After collecting one minute of baseline dynamic scans (i.e., the first eight time points), 10 mL of Gadavist (Bayer, Whippany, NJ, USA) was delivered at 2 mL/sec followed by a saline flush through a catheter placed within an antecubital vein. A population averaged arterial input function was established from the present dataset based on previously established methodology [7]. To determine the tumor ROIs, a conservative bounding-box was manually drawn over each focal lesion using the percent enhancement map (increase over 50% compared to baseline signal intensity) obtained from the DCE-MRI data. These ROIs were then refined using a fuzzy c-means clustering algorithm [28]. Going forward, we will refer to these data as the single-site dataset.

### 2.3. DCE-MRI Data Analysis

Fifteen representative patients were chosen from both the single-site and the ACRIN datasets for a total of 30 DCE-MRI patient datasets to be analyzed using two models: the Standard Kety–Tofts (SKT) model [19] and the Patlak model [23]. Due to the absence of an arterial input function for the ACRIN dataset, we employed reference regions modifications of the SKT and Patlak models to analyze the entire 30 patient dataset. The volume transfer constant (*K^trans^*) characterizes the delivery and retention of contrast agent in both the SKT and Patlak models, while the extravascular extracellular volume fraction (*v_e_*) is exclusive to the SKT models.

The full time courses (FTCs) (N = 30) were retrospectively truncated into a series of abbreviated time courses (ATCs). An ATC containing the first *n* post-injection time points of a DCE-MRI time course is referred to as “ATC*_n_*”. For application of the SKT model to the ACRIN dataset, *n* was selected as the inclusive set of integers from 7 to 18, incrementing by one (an increment of 0.25 min); for the single-site dataset, *n* was chosen to be the inclusive set ranging from 13 through 53, incrementing by eight (an increment of 1.0 min) to span the entirety of the eight-minute FTC. For the Patlak analysis of the ACRIN dataset, *n* was chosen as the inclusive set of integers from 2 to 7, incrementing by one (an increment of 0.25 min); and, for the single-site dataset, *n* was chosen as the inclusive set of integers from 5 to 14, incrementing by one (an increment of 0.12 min). Because the Patlak model assumes no washout occurs in the early part of perfusion, the range of *n* for the Patlak analysis of both datasets was chosen to include the enhancement phase across varying ATCs with an effort to exclude the washout phase entirely. The SKT and Patlak models were fit to the FTCs, as well as the ATCs, to estimate *K^trans^* (SKT and Patlak) and *v_e_* (SKT only) using the “lsqnonlin” function implemented in MATLAB (Mathworks, Natick, MA). Voxels for which the estimated parameters fell outside of the physiological range (the range being 0.001 < *K^trans^* < 5.0 and 0.001 < *v_e_* < 1.0) were eliminated from further analysis. The FTC parameter estimates were considered to be the gold standard to which all ATC*_n_* parameter estimates were compared on a voxel-wise basis.

### 2.4. DCE-MRI Simulated Data Analysis

To systematically determine errors resulting from a quantitative analysis of a truncated DCE-MRI time course, we simulated data based on the SKT model and the details of each of the 30 patients described in the previous sections. To construct such data, we started with the pulse-sequence and the *K^trans^* and *v_e_* FTC parameter values from each voxel within each patient’s acquisition to construct a set of zero-noise DCE-MRI time courses via the SKT model. Next, the signal-to-noise ratio (SNR) from each patient’s DCE-MRI study was calculated using the first seven pre-contrast time points from the adipose for the single-site dataset and the first seven time points from the adipose tissue for the ACRIN dataset. Finally, the voxel-wise SNR was averaged over the tumor ROI for the entire cohort such that each patient dataset is characterized by a single SNR value (Table 1). The ATC*_n_* of the simulated data were generated in the same fashion as in the experimental data through truncation of the simulated FTC data. The SKT and Patlak models were then fit to the both the noiseless and noisy versions of each simulated DCE-MRI time course to arrive at *K^trans^* and *v_e_* values for each voxel in the simulated tumor ROI. Again, the FTC parameter estimates were treated as the gold standard to which all ATC*_n_* parameters were compared.

### 2.5. Statistical Analysis

For all 30 patient datasets and all 30 simulated datasets, the voxel values of *K^trans^* (from both the SKT and Patlak models) and *v_e_* (SKT model only) estimated from fitting the FTCs and ATCs were averaged over the ROI to produce mean values and 95% confidence intervals (CIs) for each patient. Additionally, the absolute average percent error between the ATC*_n_* and FTC parameter values were computed and averaged over the tumor ROI along with their 95% CIs over the ACRIN and single-site patient datasets, respectively. To determine the similarity between the ATC*_n_* and FTC (gold-standard) parameter values for each voxel within the tumor ROIs, the concordance correlation coefficient (CCC, ranging from 0 to 1) was used to assess the level of agreement between each FTC–ATC*_n_* pair from each patient dataset. The Pearson’s linear correlation coefficient (*r*, ranging from −1 to 1) was used as a measure of goodness of fit for all models to all FTC and ATC*_n_* data.

Please note that the clinical methods were presented before the simulation methods to allow for a cleaner exposition of the simulation as it was based on the clinical imaging methods. In the Results section, however, we present the simulation results first so that we can then directly compare their predictions to the results of the clinical analysis.

## 3. Results

First, we examine the results from the pharmacokinetic analysis related to the ACRIN data. The SKT model was fit to one FTC and 12 ATC*_n_*s, yielding 13 sets of mean *K^trans^* and *v_e_* parameter values and the corresponding 95% CIs. Similarly, the Patlak analysis of these same patients, one FTC and six ATC*_n_*s, were analyzed to yield seven mean *K^trans^* parameter values and the corresponding 95% CIs. An analogous set of results was computed from the 15 sets of simulated data based on patient-specific signal-to-noise (SNR) values and imaging parameters of the ACRIN acquisition protocol.

### 3.1. Pharmacokinetic Assessment of ACRIN-Based Simulated Data

**Figure 1:**
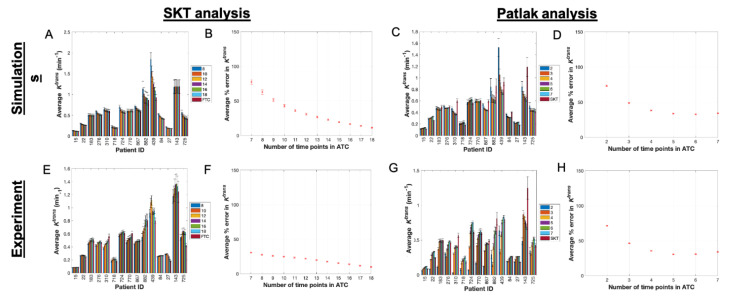
SKT and Patlak analysis of ACRIN-based simulated data and ACRIN clinical data. (**A**) Mean and 95% confidence intervals (CI) for simulated *K^trans^* values from the SKT model with the ATC length (denoted in legend) increasing from left to right in the bar plots for each patient (only a subset of all ATCs are displayed for simplicity in viewing). (**B**) Average percent error in *K^trans^* as a function of ATC length with 95% CIs. (**C**,**D**) present the analogous data for the Patlak analysis of the simulated data. The absolute error in (**B**,**D**) decreases as the ATCs are increased, but only up to a certain ATC in panel D at ATC_6_. (**E**) Mean and 95% confidence intervals (CI) for *K^trans^* values from analyzing the clinical data with the SKT model (only a subset of all ATCs are displayed for clarity). (**F**) Average percent error in *K^trans^* as a function of ATC length with 95% CIs. (**G**,**H**) present the analogous data for the Patlak analysis of the clinical data and, similar to (**D**), (**H**) shows the absolute error in *K^trans^* decreasing up to ATC_5_ before increasing again.

For each ATC*_n_* simulated from each patient, a pair of ROI-averaged *K^trans^* (Figure 1A) and *v_e_* (Figure S1A) values were compared to the corresponding FTC average parameter values obtained from the SKT model. In all simulated patients, the mean estimates of *K^trans^* and *v_e_* tended to be greater than their FTC “gold-standard” counterparts (*p* > 0.05 for all ATCs except ATC_7_, ATC_8_, and ATC_9_ for *v_e_ only*, where *p* < 0.05), revealing a systematic overestimation of both parameters as the time series were increasingly truncated. A direct relationship was observed in the CCC values of both parameters (Figures S1B and S2A), which asymptotically approached a value of 1.0 as the ATCs were lengthened. Choosing a CCC cut-off value of 0.90 for *K^trans^*, we observed that 14 patients met this cut-off for ATC_15_ (i.e., 3.8 min of scan time), with the mean and standard deviation of the CCCs being 0.94 ± 0.07. Choosing a less conservative CCC value of 0.80 as the cut-off, then the shortest ATC for which all patients met this cut-off increased to ATC_17_ (i.e., 4.25 min of scan time) with mean and standard deviation of 0.97 ± 0.05. The average percent error between the ATC*_n_*s and FTC *K^trans^* values monotonically decreased with longer ATCs (e.g., 11.30% error for ATC_18_ compared to 77.25% error for ATC_7_) (Figure 1B), though the percent error was significantly higher (*p* < 0.05) in *v_e_* than in *K^trans^* (44.60% error for ATC_18_ compared to 106.68% error for ATC_7_) (Supplemental Figure S1C) over all ATC*_n_* lengths.

Next, we look at the corresponding results from the Patlak analysis. In nearly all simulated patients, the mean estimates of *K^trans^* (Figure 1C) from all ATC*_n_*s were closer in value to their FTC “gold-standard” counterparts as the time series were increasingly truncated. The CCCs (Supplemental Figure S2B) did not monotonically increase toward a value of 1.0 for all patients, instead more often peaking at a specific ATC*_n_* before decreasing again; and two patients exhibited monotonically decreasing CCCs. Choosing a CCC cut-off value of 0.90 for *K^trans^* yielded a maximum of two patients that meet this cut-off for ATC_3_ (i.e., 0.75 min of scan time), with the mean and standard deviation of the CCCs being 0.75 ± 0.13. For a less conservative CCC value of 0.80 as the cut-off, a maximum of seven patients met this cut-off at ATC_4_ (i.e., 1.0 min of scan time) with a mean and standard deviation of 0.76 ± 0.14. The average percent error between the ATC*_n_* and FTC *K^trans^* values monotonically decreased until a minimum was reached at ATC_6_ (32.66% error) before increasing again with ATC_7_ (34.18% error) (Figure 1D).

### 3.2. Pharmacokinetic Assessment of ACRIN Clinical Data

**Figure 2:**
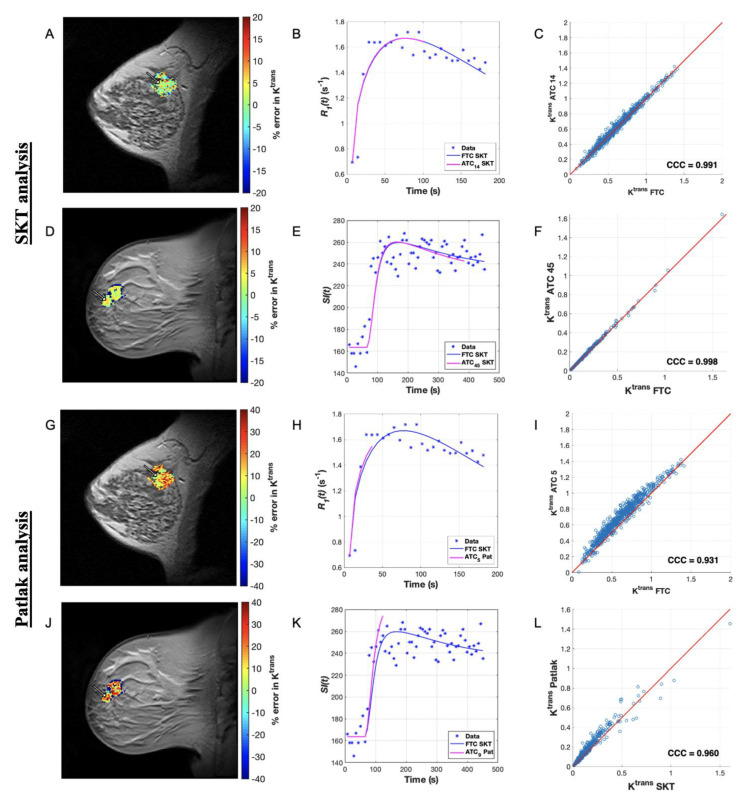
Comparing SKT and Patlak *K^trans^* error for a long ATC for a representative patient from each dataset. (**A**) Map of percent error in *K^trans^* over the tumor ROI for an ACRIN patient dataset analyzed with the SKT model for ATC_14_ (3.5 min). (**B**) Plot of longitudinal relaxation rate curves, *R_1_*(*t*), for a representative voxel as indicated by the arrow in (**A**) with curves labeled in the legend. (**C**) Scatter plot of *K^trans^* ATC_14_ and *K^trans^* FTC values in the ROI, where the line of unity is in red. (**D**) Map of percent error in *K^trans^* over the tumor ROI for a single-site patient dataset analyzed with the SKT model for ATC_45_ (5.45 min). (**E**) Plot of signal intensity curves, *SI*(*t*), for a representative voxel indicated by the arrow in (**D**) with curves labeled in the legend. (**F**) Scatter plot of *K^trans^* ATC_45_ and *K^trans^* FTC values in the ROI, where line of unity is in red. (**G**–**L**) present the analogous data for the Patlak analysis for ATC_5_ (1.25 min) and ATC_9_ (1.09 min) from the ACRIN and single-site datasets, respectively. We observe generally close fits in (**B**,**E**) as well as high agreement (CCCs > 0.80) in the FTC and ATC parameters in (**C**,**F**) as well as in the analogous Patlak fits in (**H**,**K**) and the Patlak parameters in (**I**,**L**).

**Figure 3:**
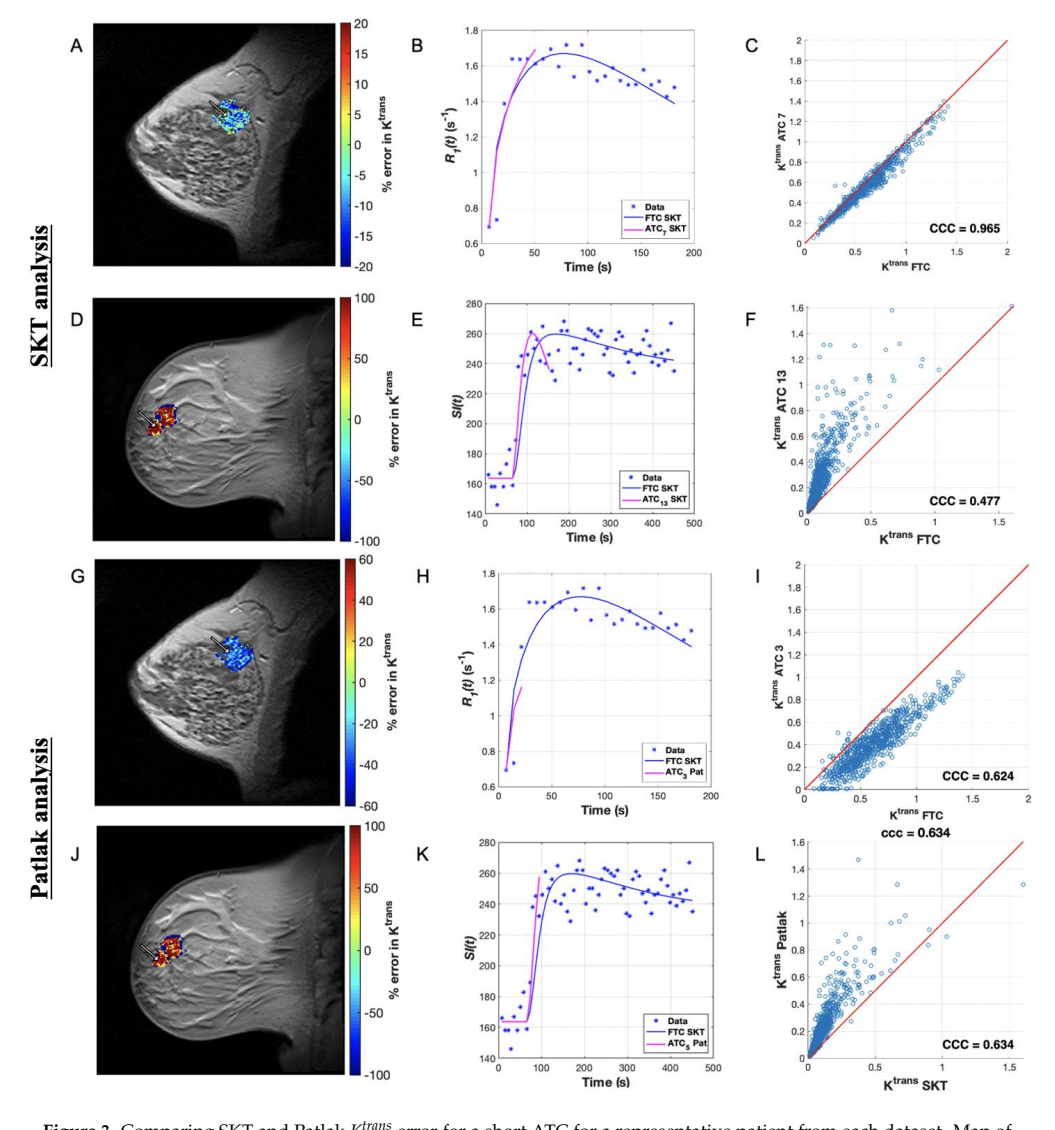
Comparing SKT and Patlak *K^trans^* error for a short ATC for a representative patient from each dataset. Map of percent error in *K^trans^* over the tumor ROI for an ACRIN patient analyzed with the SKT model for ATC_7_ (1.75 min). (**B**) Plot of longitudinal relaxation rate curves, *R_1_*(*t*), for a representative voxel indicated by the arrow in (**A**) with curves labeled in the legend. (**C**) Scatter plot of *K^trans^* ATC_7_ and *K^trans^* FTC values in the ROI, where the line of unity is in red. (**D**) Map of percent error in *K^trans^* over the tumor ROI for a single-site patient analyzed with the SKT model for ATC_13_ (1.60 min). (**E**) Plot of signal intensity curves, *SI*(*t*), for a representative voxel indicated by the arrow in (**D**) with curves labeled in the legend. (**F**) Scatter plot of *K^trans^* ATC_13_ and *K^trans^* FTC values in the ROI, where line of unity is in red. (**G**–**L**) present the analogous data for the Patlak analysis for ATC_3_ (0.75 min) and ATC_5_ (0.61 min) from the ACRIN and single-site datasets, respectively. We observe generally poorer fits in (**B**,**E**) as well as less agreement (CCC < 0.80) in the FTC and ATC parameters in (**C**,**F**) as well as in the analogous Patlak fits in (**H**,**K**) and the Patlak parameters in (**I**,**L**).

After examining the simulated data, we applied the same analysis with the SKT model to the ACRIN clinical data. The mean estimates of *K^trans^* (Figure 1E) and *v_e_* (Figure S1D) from all ATC*_n_*s were not significantly different from their FTC “gold-standard” counterparts (*p* > 0.05 for all ATCs except ATC_7_, ATC_8_, and ATC_7_ for *v_e_ only*, where *p* < 0.05), revealing a systematic underestimation in *K^trans^* and overestimation in *v_e_* as the time series were increasingly truncated (Figure 2A–C and Figure 3A–C, Figures S5A–C and S6A–C). A direct relationship was observed in the CCC values of both parameters (Figures S1E and S2C), which asymptotically approached a value of 1.0 as the lengths of the ATC*_n_*s were increased. Choosing a CCC cut-off value of 0.90 for *K^trans^*, we observed that at most 11 patients met this cut-off for ATC_14_ (i.e., 3.5 min of scan time) with CCCs of 0.88 ± 0.16. If we choose a less conservative CCC value of 0.80 as the cut-off, then the shortest ATC*_n_* for which a maximum of 12 patients met this cut-off is again ATC_14_ (i.e., 3.5 min of scan time). The average percent error between the ATC*_n_*s and FTC *K^trans^* values monotonically decreased with longer ATC*_n_*s (9.77% error for ATC_18_ compared to 30.60% error for ATC_7_) (Figure 1F), though the percent error was higher (*p* = 0.08) in *v_e_* than in *K^trans^* over nearly all ATC*_n_* lengths except ATC_18_ (9.16% error for ATC_18_ compared to 106.61% error for ATC_7_) (Supplemental Figure S1F).

The Patlak analysis of these same data reveal that in nearly all patient datasets, the mean estimates of *K^trans^* (Figure 1G) from all ATC*_n_*s approached their FTC “gold-standard” counterparts for intermediary abbreviations rather than the shortest or longest ones (Figure 2G–I and Figure 3G–I). The CCCs (Figure S2D) did not monotonically increase toward a value of 1.0 for all patients, instead more often peaking at a specific ATC*_n_* before fluctuating in value thereafter. Choosing a CCC cut-off value of 0.90, a maximum of four patients met this cut-off for ATC_4_ (i.e., 1.0 min of scan time) with mean and standard deviation of the CCCs being of 0.74 ± 0.21. For a CCC cut-off value of 0.80, the shortest ATC for which a maximum number of patients, namely eight, met this CCC cut-off was ATC_5_ (i.e., 1.25 min of scan time) with a mean and standard deviation of 0.77 ± 0.18. The average percent error between the ATC and FTC *K^trans^* values monotonically decreased until a minimum was reached at ATC_5_ (30.51% error) before increasing again with ATC_6_ (30.79% error) (Figure 1H).

We now turn to the pharmacokinetic analysis of the 15 single-site patient datasets. The SKT model was fit to one FTC and six ATC*_n_*s, yielding seven sets of mean *K^trans^* and *v_e_* parameter values and the corresponding 95% CIs. Similarly, for analysis with the Patlak model of these same patients, one FTC and ten ATC*_n_*s were analyzed, yielding eleven mean *K^trans^* parameter values and the corresponding 95% CIs. An analogous set of results was computed from the 15 sets of simulated data based on patient-specific SNR values and the imaging parameters of the single-site acquisition protocol.

### 3.3. Pharmacokinetic Assessment of Single-Site-Based Simulated Data

**Figure 4:**
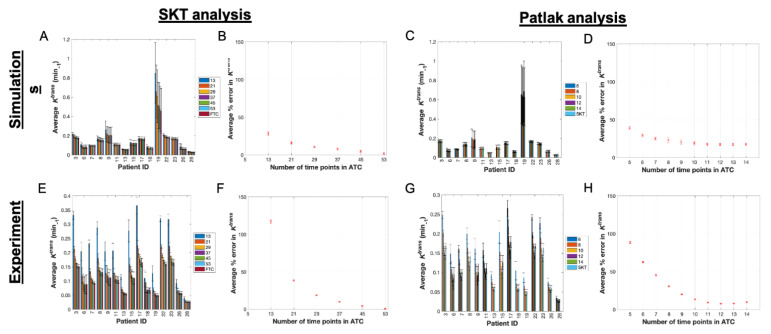
SKT and Patlak analysis of single-site-based simulated data and single-site clinical data. (**A**) Mean and 95% confidence intervals (CI) for simulated *K^trans^* values from the SKT model with the ATC length (denoted in legend) increasing from left to right in the bar plots for each patient (only a subset of all ATCs are displayed for clarity). (**B**) Average percent error in *K^trans^* as a function of ATC length with 95% CIs. (**C**,**D**) present the analogous data for the Patlak analysis of the simulated data. The absolute error in (**B**,**D**) decreases as the ATCs are increased, but only up to a certain ATC in panel D at ATC_12_. (**E**) Mean and 95% confidence intervals (CI) for *K^trans^* values from analyzing the clinical data with the SKT model (only a subset of all ATCs are displayed for simplicity in viewing) (**F**) Average percent error in *K^trans^* as a function of ATC length with 95% CIs. (**G**,**H**) present the analogous data for the Patlak analysis of the clinical data and, similar to (**D**), (**H**) shows the absolute error in *K^trans^* decreasing up to ATC_12_ before increasing again.

By fitting the SKT model to the simulated patients, we find that the mean estimates of *K^trans^* (Figure 4A) and *v_e_* (Figure S3A) from all ATC*_n_*s tented to be greater than their FTC “gold-standard” counterparts (*p* > 0.05). A direct relationship was observed in the CCC values of both parameters (Figures S3B and S4A), which asymptotically approached a value of 1.0 as the ATCs were lengthened. Choosing a CCC cut-off value of 0.90 for *K^trans^*, we observed that all ten patients met this cut-off for ATC_37_ (i.e., 4.5 min of scan time) with the mean and standard deviation of the CCCs being 0.99 ± 0.02. If we choose a less conservative CCC value of 0.80 as the cut-off, then the shortest ATC for which all patients meet this cut-off is ATC_29_ (i.e., 3.5 min of scan time) with a mean and standard deviation of 0.98 ± 0.02. The average percent error between the ATC*_n_*s and FTC *K^trans^* values monotonically decreased with longer ATCs (1.93% error for ATC_53_ compared to 28.51% error for ATC_13_) (Figure 4B). This percent error was systematically higher (*p* = 0.07), in *v_e_* (4.23% error for ATC_53_ compared to 135.13% error for ATC_13_) (Figure S3C) than in *K^trans^* over the course of all ATC*_n_* lengths.

Applying the Patlak model in an analogous manner, we find that the mean estimates of *K^trans^* (Figure 4C) from all ATCs were closer in value to their FTC “gold-standard” counterparts as the time series were increasingly abbreviated. The CCCs (Figure S4B) did not monotonically increase toward a value of 1.0 for all patients, instead more often peaking at a specific ATC*_n_* before fluctuating in value thereafter. Choosing a CCC cut-off value of 0.90 for *K^trans^*, we observe that a maximum of nine patients met this cut-off for ATC_9_ (i.e., 1.09 min of scan time) with CCCs of 0.88 ± 0.10. If we choose a less conservative CCC value of 0.80 as the cut-off, then the shortest ATC*_n_* for which a maximum of 13 patients met this CCC cut-off was ATC_6_ (i.e., 0.73 min of scan time). The average percent error between the ATC*_n_*s and FTC *K^trans^* values monotonically decreased until a minimum was reached at ATC_12_ (17.34% error) before increasing again with ATC_13_ (17.40% error) (Figure 4D).

### 3.4. Pharmacokinetic Assessment of Single-Site Clinical Data

Next, we summarize the SKT analysis of the experimental data. In all patient datasets, the mean estimates of *K^trans^* (Figure 4E) and *v_e_* (Figure S3D) from all ATC*_n_*s were, respectively, greater than and less than (*p* > 0.05) their FTC “gold-standard” counterparts (except for significance in ATC_13_ for *K^trans^* where *p* < 0.05). This reveals a systematic overestimation in *K^trans^* and underestimation in *v_e_* as the time series were increasingly truncated (Figure 2D–F and Figure 3D–F, Figures S5D–F and S6D–F). A direct relationship was observed in the CCC values of both parameters (Figures S3E and S4C), which asymptotically approached a value of 1.0 as the lengths of the ATC*_n_*s were lengthened. Choosing a CCC cut-off value of 0.90 for *K^trans^*, all ten patients met this cut-off for ATC_37_ (i.e., 4.5 min of scan time) with the mean and standard deviation of the CCCs being 0.99 ± 0.02. If we choose a less conservative CCC value of 0.80 as the cut-off, then the shortest ATC for which 14 out of 15 patients met this CCC cut-off was ATC_29_ (i.e., 3.5 min of scan time) with a mean and standard deviation of 0.94 ± 0.04. The average percent error between the ATC*_n_* and FTC *K^trans^* values decreased monotonically with longer ATC*_n_*s (0.63% error for ATC_53_ compared to 117.34% error for ATC_13_) (Figure 4F), though the percent error was higher (*p* = 0.59) in *v_e_* than in *K^trans^* over the course of nearly all ATC lengths except ATC_13_ (2.19% error for ATC_53_ compared to 74.27% error for ATC_13_) (Figure S3F).

Applying the Patlak model in an analogous manner, we find that the mean estimates of *K^trans^* (Figure 4G) from all ATCs deviated from their FTC “gold-standard” counterparts in nearly all simulated patients as the time series were increasingly abbreviated, with significant differences observed in ATC_5_ (*p* = 0.005) (Figure 2J-L and Figure 3J–L). The CCCs (Figure S4D) were again observed to not monotonically increase toward a value of 1.0 for all patients, instead more often peaking at a specific ATC before fluctuating in value thereafter. Choosing a CCC cut-off value of 0.90, we observed that at most 12 patients meet this cut-off for ATC_10_ (i.e., 1.20 min of scan time) from the set of CCCs with a mean and standard deviation of 0.91 ± 0.09. If we choose a less conservative CCC value of 0.80 as the cut-off, then the shortest ATC*_n_* for which a maximum of 12 patients meets this CCC cut-off is ATC_10_ again. The average percent error between the ATC and FTC *K^trans^* values decreased monotonically until a minimum was reached at ATC_12_ (7.67% error) before increasing again with ATC_13_ (7.95% error) (Figure 4H).

## 4. Discussion

To the best of our knowledge, this work provides the first quantitative characterization of the errors associated with a pharmacokinetic analysis of abbreviated DCE-MRI time courses. The primary finding of this study showed that *K^trans^* exhibits substantially low error and high CCC values across ATCs for both the SKT and Patlak analyses. This strongly suggests that the length of a DCE-MRI measurement can be substantially shortened without a substantial reduction in the ability to quantify the pharmacokinetics. Our results indicate it is feasible for 80% of patients from the single-site cohort analyzed by the Patlak model to exceed a CCC for *K^trans^* of 0.80 for an abbreviated time course as short as 1.20 min. Similarly, it is feasible for 60% of patients from the ACRIN cohort (with a substantially poorer temporal resolution) analyzed by the Patlak model to exceed a CCC for *K^trans^* of 0.80 for an abbreviated time course as short as 1.25 min. This implies that abbreviated—but still quantitative—DCE-MRI can be performed for screening high-risk patients. This reduction in total scan time can then be “spent” on making additional measurements of interest (e.g., diffusion-weighted MRI [29,30]), or simply be used to shorten the entire examination. In particular, *K^trans^* may add specificity in distinguishing malignant lesions in DCE-MRI screening scans for high-risk women [13]. Conversely, as *v_e_* has not yet been shown to statistically separate malignant from benign tissue, collecting the full extent of the washout phase may not be necessary. It is important to recall that this study also made use of data acquired in two very different settings: a multi-site, clinical trial run at academic research-oriented medical centers, and a single-site, community-based care setting. Thus, the results have the potential to be generalizable across clinical imaging environments.

As a larger portion of the washout phase of the DCE-MRI time course was excluded from the single-site and ACRIN patient datasets, the absolute error in *v_e_* from the SKT model was consistently higher than the absolute error in *K^trans^* across most ATC*_n_*s when compared to the FTC. *K^trans^* and *v_e_* are largely determined by the enhancement and washout phases, respectively; thus, as the data truncation did not exclude the enhancement phase, a smaller absolute error is expected in *K^trans^* than in *v_e_*. In terms of CCCs from the SKT analysis, we found that a similar number of patients met the higher CCC cut-off for *K^trans^* of 0.90 in the single-site cohort (93% in the experimental data analysis for a 3.5 min abbreviation, 100% in the simulation data analysis for a 3.5 min abbreviation) and the ACRIN cohort (80% in the experimental data analysis for a 4.25 min abbreviation, 100% in the simulation data analysis for a 3.5 min abbreviation). The Patlak analysis of the single-site cohort achieved smaller absolute error in both the simulated (17.34% error for a 1.5 min abbreviation) and experimental (7.67% error for a 1.5 min abbreviation) datasets compared to the Patlak analysis of the ACRIN cohort in both the simulated (32.66% error for a 1.25 min abbreviation) and experimental (30.51% error for a 1.5 min abbreviation) datasets. This difference is most likely due to the superior temporal resolution of the single-site study compared to the ACRIN study (7.27 versus 15 s). In terms of CCCs from the Patlak analysis, we found that more patients met the higher CCC cut-off of 0.90 in the single-site cohort (80% in the experimental data analysis for a 1.2 min abbreviation, 60% in the simulation data analysis for 1.2 and 1.09 min abbreviations, respectively) than the ACRIN cohort did (27% in the experimental data analysis for a 1.0 min abbreviation, 13% in the simulation data analysis for a 0.75 min abbreviation). The Patlak model will improve in accuracy as long as the amount of data from the enhancement phase of the DCE-MRI time course is being increased; but once the data begins to include the plateauing and washout phases of the time course, the Patlak model is no longer an appropriate model, and its accuracy in parameter estimation begins to decrease. Overall, these findings indicate that an abbreviated DCE-MRI breast scan with sufficient temporal resolution can be feasibly analyzed with the Patlak model as well as the SKT model to produce *K^trans^* values that closely match those from analyzing a full-length scan with the SKT model.

While the SKT and Patlak model analysis is amendable to quantitative DCE-MRI scans, clinicians in practice rely on the semi-quantitative signal enhancement ratio (SER) that is often computed using an image captured at the end of a clinical DCE-MRI scan. While we do not consider this measure in our abbreviated study, it remains possible that the SER may be computed by capturing a final washout image after the abbreviated quantitative DCE-MRI protocol and any additional scans are completed within the standard time of a clinical scan.

There are multiple opportunities to strengthen the results of this study. For instance, it has been shown that incorporation of intra-voxel diffusion into DCE-MRI models leads to more accurate estimation of pharmacokinetic parameters [31]; therefore, it may be of interest to investigate the effect of contrast agent diffusion on the DCE-MRI data acquired in the abbreviated setting. To perform such an analysis would require higher temporal resolution data, though compared to the high spatial resolution DCE-MRI scans acquired in the standard-of-scare setting, the datasets in this study already have much higher temporal resolution. Increasing the temporal resolution further to characterize the diffusion phenomenon may potentially limit the ability to translate abbreviated, quantitative DCE-MRI to widespread clinical application since an increase in temporal resolution would lead to a further decrease in spatial resolution. Similarly, the lower SNR associated with high temporal resolution DCE-MRI data also makes application in standard radiological practice less attractive. Another limitation is that it is very difficult to directly compare the results obtained from the two datasets utilized in this study due to the differences in their respective imaging protocols including, in particular, the flip angles of 6° and 90° for the single-site and ACRIN protocols, respectively. The DCE-MRI time course data from the patients scanned at the single-site, community setting typically reached a saturated signal intensity, making it difficult to accurately quantify *v_e_*; this can be remedied by employing a larger flip angle. Conversely, the 90° flip angle employed in the ACRIN study will limit the image contrast and reduce the overall SNR (being far away from the Ernst angle). In addition, the presence and location of breast clips post-biopsy and how they affected the signal intensity curves in the surrounding tissue were not available and thus not considered in the perfusion model analyses [32]. Lastly, while strides have been made toward reproducible quantitative DCE-MRI of the breast across multiple sites [33,34], the results of the present study could be strengthened by being repeated in prospectively abbreviated quantitative scans with uniform imaging parameters across multiple sites.

In summary, this work quantifies the errors introduced in pharmacokinetic model parameters as a function of the length of the time series for two distinct quantitative DCE-MRI datasets with different temporal resolutions. The ability to compute *K^trans^* in the abbreviated setting has shown promise with 100% of patients meeting a stringent CCC cut-off of 0.90 for *K^trans^* from the SKT analysis in the single-site cohort for a 4.5 min abbreviation and at least 73% of patients from the ACRIN cohort for a 3.5 min abbreviation). At least 80% of patients met a stringent CCC cut-off of 0.90 from the Patlak analysis for the single-site cohort. These robust results indicate the potential for employing abbreviated quantitative DCE-MRI scans for screening high-risk patients in the routine clinical setting.

## Figures and Tables

**Table 1:** Summary of the ACRIN (top sub-table) and single-site (bottom sub-table) datasets.

Patient			Site				Length			SNR			Diagnosis (benign = 0/malig = 1)		
15			1				19			14			0		
22			1				24			22			0		
183			1				24			26			1		
276			1				27			26			0		
310			1				27			24			1		
718			1				27			25			0		
724			3				25			16			1		
770			1				31			30			1		
867			2				22			18			1		
882			2				22			22			1		
439			3				25			22			0		
84			1				20			13			1		
27			1				21			28			0		
143			1				24			33			1		
725			1				29			6			0		
**Patient**	3	6	7	8	9	11	13	15	17	18	19	22	23	26	28
**SNR**	19	19	26	19	14	27	24	21	26	19	8	21	25	22	27

## Data Availability

The single-site datasets presented in this study are available on request from the corresponding author and are not publicly available due to extra precaution with any potential protected health information as well as the fact that data collection is ongoing. The multi-site data from the IBMC 6883 protocol is available on request from ACRIN.

## Supplementary Materials

The following are available online at https://www.mdpi.com/article/10.3390/tomography7030023/s1: Figure S1: Analysis of *v_e_* from fitting the SKT model to ACRIN-based simulated data and ACRIN clinical data, Figure S2: CCCs from SKT and Patlak model analysis of ACRIN-based simulated data and ACRIN clinical data, Figure S3: Analysis of *v_e_* from fitting the SKT model to single-site-based simulated data and single-site clinical data, Figure S4: CCCs from SKT and Patlak model analysis of single-site-based simulated data and single-site clinical data., Figure S5: Comparing SKT ve error for a long ATC for a representative patient from each dataset, and Figure S6: Comparing SKT *v_e_* error for a short ATC for a representative patient from each dataset.

## Abbreviations

DCE-MRI	dynamic contrast-enhanced MRI
FTC	full time course
ATC	abbreviated time course
GRAPPA	generalized autocalibrating partial parallel acquisition
BI-RADS	breast imaging reporting and data system
IBMC	International Breast MR Consortium
ACRIN	American College of Radiology Imaging Network
ROI	region of interest
SPGR	spoiled gradient echo
