# Supplementary material for: Characterizing Errors in Pharmacokinetic Parameters from Analyzing Quantitative Abbreviated DCE-MRI Data in Breast Cancer
Source: Tomography. 2021 Jun 23;7(3):253–67. doi: 10.3390/tomography7030023 (PMC8293327; doi:10.3390/tomography7030023)
Supplement: Supplementary file 1 [file tomography-07-00023-s001.zip › tomography-1173079-supplementary.pdf]

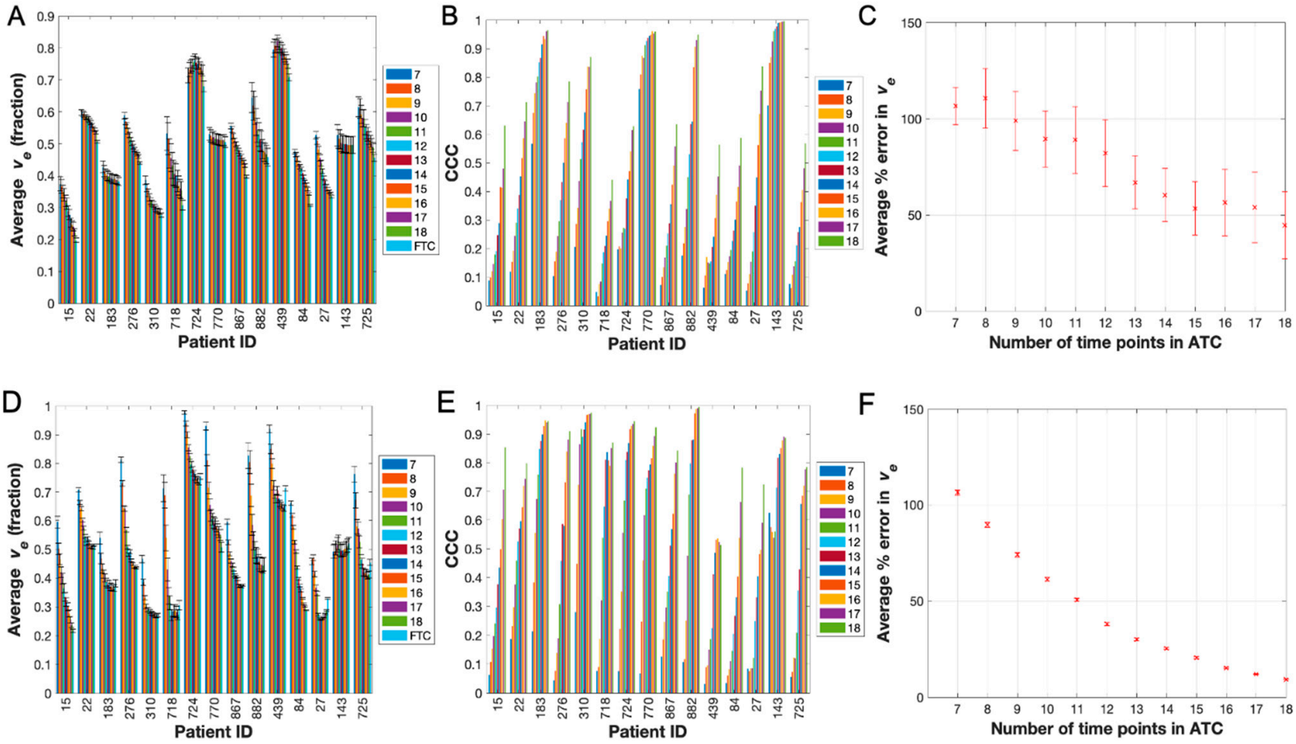

Figure S1. Analysis of v_e from fitting the SKT model to ACRIN-based simulated data and ACRIN clinical data.

(**A**) Mean and 95% confidence intervals (CI) for v_e values for ACRIN-based simulated patient datasets with ATC length (denoted in legend) increasing from left to right in the bar plots for each patient. (**B**) CCCs comparing the v_e FTC values and each set of v_e ATC values for the simulated data. (**C**) Average percent error in v_e as a function of ATC length with 95% CIs for the simulated data. (**D**–**F**) present the analogous results for the ACRIN clinical data. The CCCs in (**B**) and (**E**) approach the maximum value of 1.0 as the ATCs are lengthened.

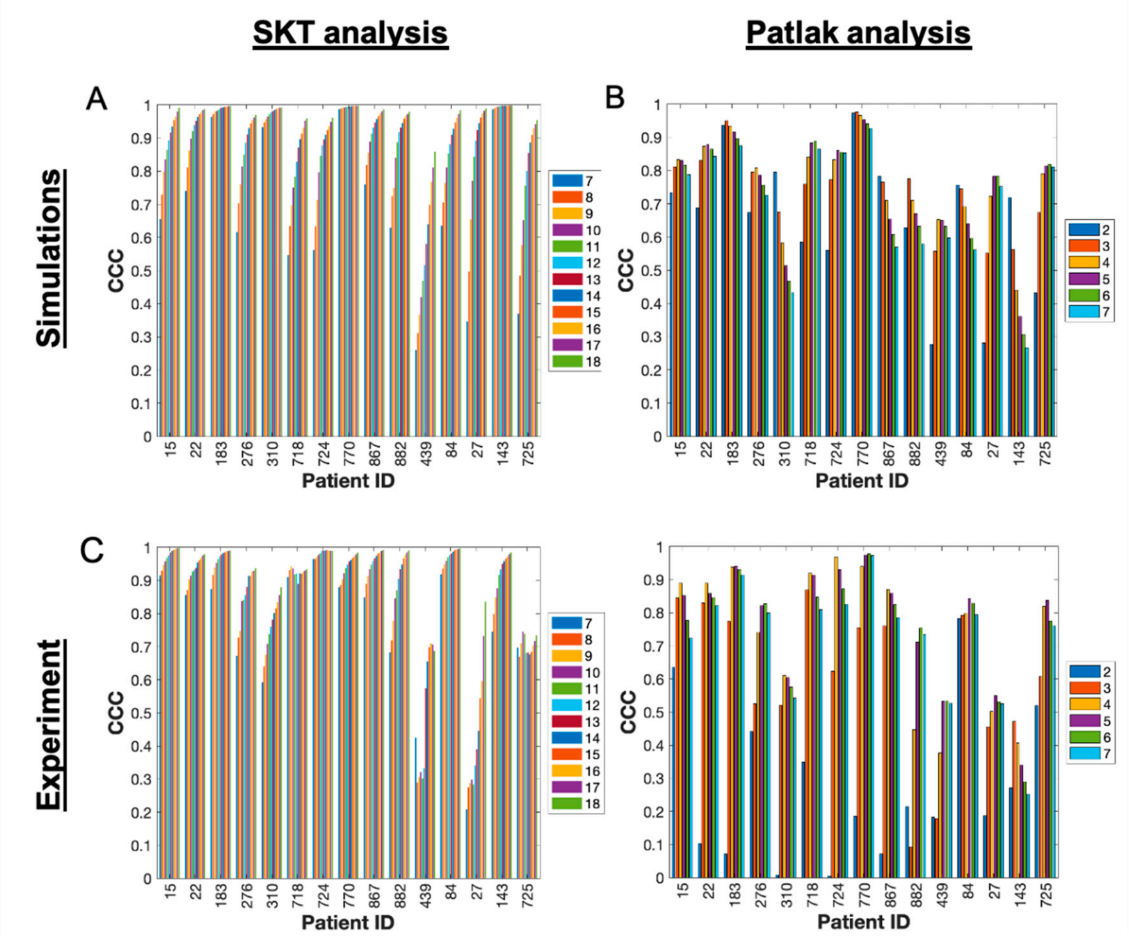

Figure S2. CCCs from SKT and Patlak model analysis of ACRIN-based simulated data and ACRIN clinical data.

(A) Concordance correlation coefficients (CCCs) comparing the simulated K^{trans} FTC values and each set of ACRIN simulation-based K^{trans} ATC values from SKT model analysis. (**B**) Analogue of (**A**) for the Patlak model analysis of the simulated data. (**C**) CCCs comparing the K^{trans} FTC values and each set of K^{trans} ATC values from SKT model analysis of the clinical data. (**D**) Analogue of (**C**) for the Patlak model analysis of the clinical data.

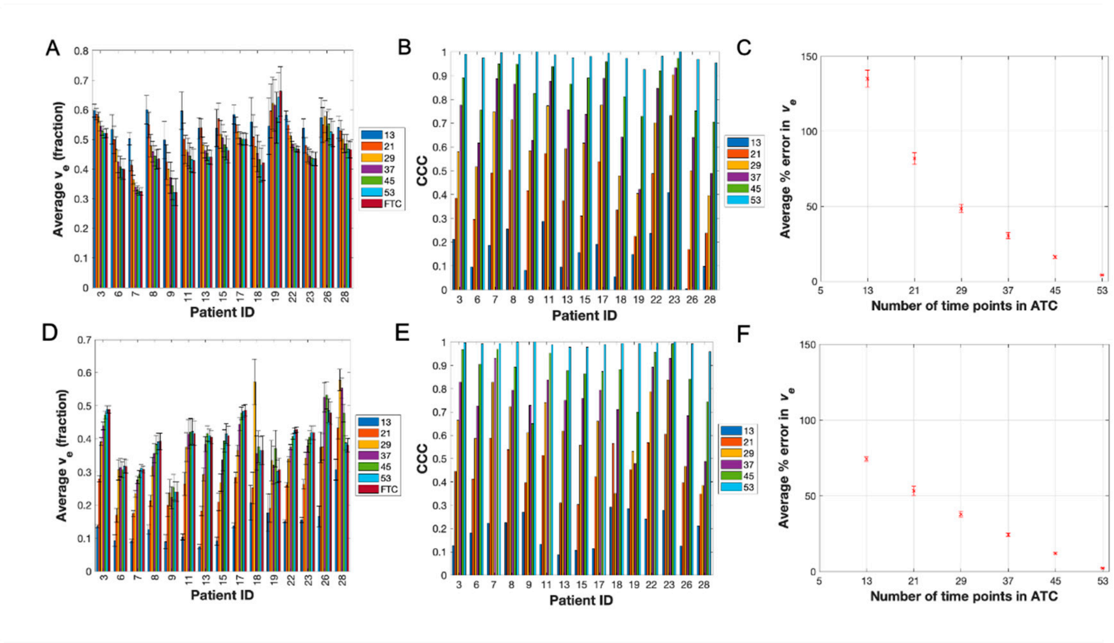

Figure S3. Analysis of v_e from fitting the SKT model to single-site-based simulated data and single-site clinical data.

(**A**) Mean and 95% confidence intervals (CI) for v_e values for single-site-based simulated patient datasets with ATC length (denoted in legend) increasing from left to right in the bar plots for each patient. (**B**) CCCs comparing the v_e FTC values and each set of v_e ATC values for the simulated data. (**C**) Average percent error in v_e as a function of ATC length with 95% CIs for the simulated data. (**D**–**F**) present the analogous results for the single-site clinical data. The CCCs in (**B**) and (**E**) approach the maximum value of 1.0 as the ATCs are lengthened.

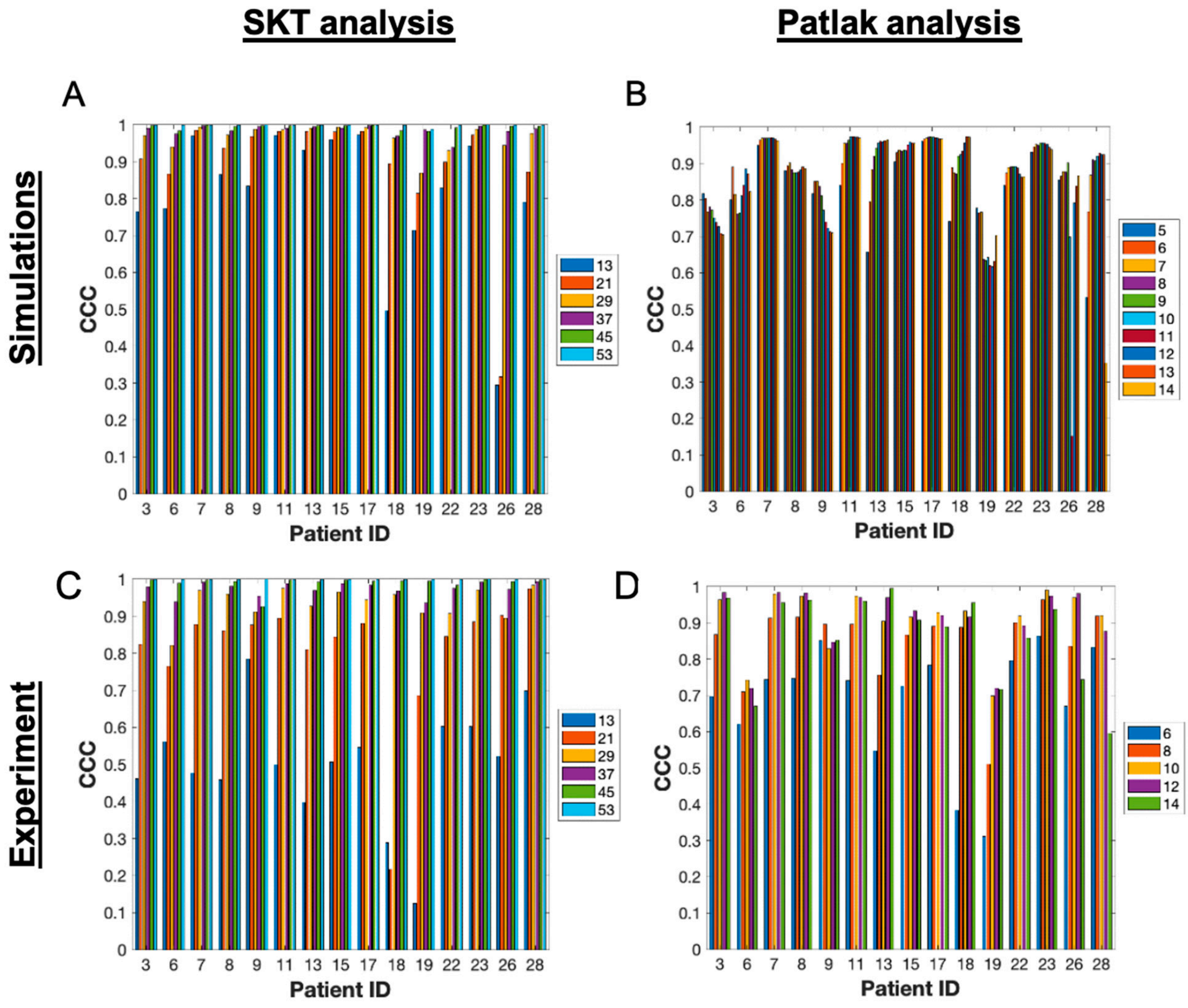

Figure S4. CCCs from SKT and Patlak model analysis of single-site-based simulated data and single-site clinical data.

(**A**) Concordance correlation coefficients (CCCs) comparing the simulated K^{trans} FTC values and each set of single-site simulation-based K^{trans} ATC values from the SKT model analysis. (**B**) Analogue of (**A**) for the Patlak model analysis of the simulated data. (**C**) CCCs comparing the K^{trans} FTC values and each set of K^{trans} ATC values from SKT model analysis of the clinical data. (**D**) Analogue of (**C**) for the Patlak model analysis of the clinical data.

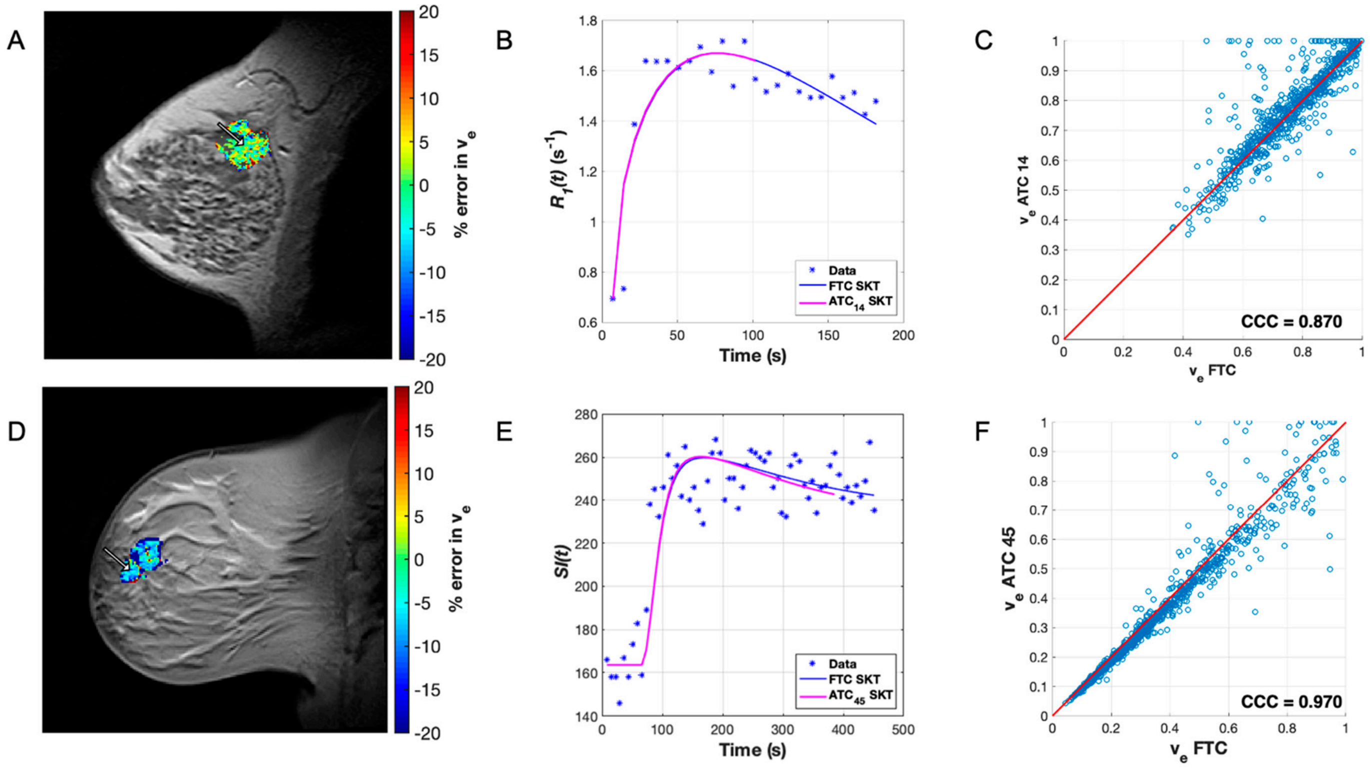

Figure S5. Comparing SKT v_e error for a long ATC for a representative patient from each dataset.

(**A**) Map of percent error in v_e over the tumor ROI for an ACRIN patient dataset analyzed with the SKT model for AT_{14} (3.5 min.). (**B**) Plot of longitudinal relaxation rate curves, $R_1(t)$, for a representative voxel as indicated by the arrow in (A) with curves labelled in the legend. (**C**) Scatter plot of v_e ATC_{14} and v_e FTC values in the ROI, where the line of unity is in red. (**D**)-(**F**) present the analogous data for the single-site dataset. We observe generally close fits in (B) and (E) as well as high agreement (CCCs > 0.80) in the FTC and ATC parameters in (C) and (F).

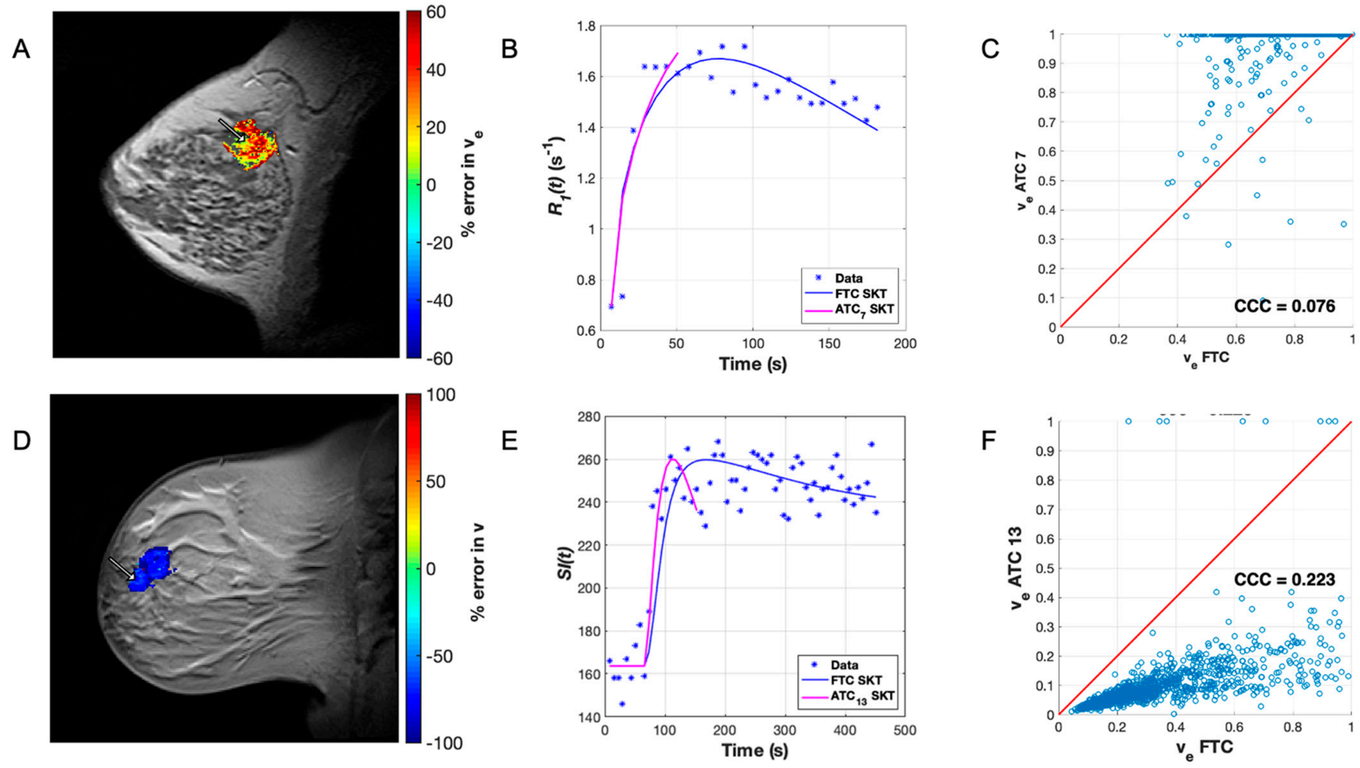

Figure S6. Comparing SKT v_e error for a short ATC for a representative patient from each dataset.

(**A**) Map of percent error in v_e over the tumor ROI for an ACRIN patient dataset analyzed with the SKT model for ATC_7 (1.75 min.). (**B**) Plot of longitudinal relaxation rate curves, $R_1(t)$, for a representative voxel as indicated by the arrow in (A) with curves labelled in the legend. (**C**) Scatter plot of v_e ATC_7 and v_e FTC values in the ROI, where the line of unity is in red. (**D**)-(**F**) present the analogous data for the single-site dataset. We observe generally close fits in (B) and (E) as well as high agreement (CCCs > 0.80) in the FTC and ATC parameters in (C) and (F).
